# Signalment and laboratory findings in cats with diabetes mellitus in Germany: a retrospective review of laboratory submissions of 129,505 cats

**DOI:** 10.1177/1098612X241262669

**Published:** 2024-09-17

**Authors:** Bente Guse, Judith Langenstein, Natali Bauer, Katarina Hazuchova

**Affiliations:** 1 Clinic for Small Animals (Internal Medicine, Clinical Pathology and Clinical Pathophysiology), Justus-Liebig-University Giessen, Germany; 2 Antech Lab Germany GmbH, Augsburg, Germany

**Keywords:** Diabetic, prevalence, risk factors, hyperlipidaemia, hyperglycaemia

## Abstract

**Objectives:**

The aims of this study were to compare signalment and laboratory parameters between diabetic (D) and non-diabetic (ND) cats and poorly-controlled diabetic (PD) and well-controlled diabetic (WD) cats in Germany.

**Methods:**

Laboratory data from Antech Lab Germany between 2015 and 2018 were retrospectively analysed. Age, sex, red blood cell count (RBC), creatinine (CREA), alkaline phosphatase (AP), alanine aminotransferase (ALT), bilirubin (BILI), cholesterol (CHOL), triglycerides (TRI), glucose (GLU) and total thyroxine (TT4) were compared between D (fructosamine ⩾340 µmol/l) and ND cats, and PD (fructosamine >500 µmol/l) and WD (fructosamine 340–500 µmol/l) cats. The proportion of cats with anaemia (RBC ⩽4.21 ×10^12^/l), CREA >250 µmol/l, ALT >455 U/l, AP >315 U/l, BILI ⩾35 µmol/l and TT4 > reference interval (RI) was compared between PD and WD cats. Data are presented as median and interquartile range (IQR) and analysed using non-parametric tests. Significance was *P*<0.05, and effect size was assessed by Cramér V or r.

**Results:**

In total, 129,505 cats were included (D: n = 9334 [prevalence 7.2%], WD: n = 5670/9334 [60.7%]). The median age of D and ND cats was 12 years (IQR D 9–14; ND 9–15); there was no difference in sex. A significant difference was found between groups (D vs ND; PD vs WD) for all parameters studied. Considering the effect sizes and medians outside the RI, the only relevant difference was higher CHOL, TRI, AP and GLU in PD compared with WD (CHOL: PD 7.46 [5.85–9.32] vs WD 5.44 [4.32–6.97] mmol/l, *P*<0.001, r = 0.39; TRI: PD 1.44 [0.84–3.66] vs WD 0.78 [0.5–1.35] mmol/l, *P* <0.001, r = 0.35; AP: PD 66 [47–92] vs WD 35 [23–59] U/l, *P* <0.001, r = 0.39; GLU: PD 23.7 [20.15–27.3] vs WD 6.89 [5–11.31] mmol/l, *P* <0.001, r = 0.69).

**Conclusions and relevance:**

Laboratory changes in diabetic cats were mild and mainly associated with lipid derangements.

## Introduction

Diabetes mellitus (DM) is one of the most common endocrinopathies in cats. Estimates of prevalence in the range of 0.08–1.24% have been reported for a few countries, such as the UK, Sweden, Australia and the USA,^1–5^ but for most countries, the prevalence is unreported. Older age (usually >11 years) has been uniformly identified as a risk factor in numerous studies.^2,4–8^ Furthermore, in most studies, the male sex was overrepresented and considered a risk factor for DM.^1,3,5,6,8–10^ In contrast, O’Neill et al^2^ could not detect a significant difference between the sexes after taking the effects of other risk factors (body weight) into account. Discrepancies between studies also exist regarding the influence of neutering. Although McCann et al^1^ detected an increased risk for neutered cats to develop DM, Prahl et al^5^ did not.

The use of different databases (first opinion practice, large hospitals, universities) and different statistical methods might explain the wide range of reported prevalences and some variation in risk factors; however, true regional differences might also exist. Given the differences in reported data across studies, knowledge of local prevalence and risk factors might be useful to veterinarians and researchers, as well as other parties involved in veterinary care (eg, insurance companies, pharmaceutical companies).

In most diabetic cats, laboratory tests are performed at the time of diagnosis and/or at follow-up visits; however, studies reporting on clinicopathological findings other than the expected hyperglycaemia and elevated fructosamine are scarce. One older investigation evaluated laboratory submissions of 104 diabetic cats between 1992 and 1994 and found that most cats with DM had hypercholesterolaemia and elevation of at least one liver enzyme.^9^ Furthermore, a review on the interpretation of clinical pathology findings in geriatric veterinary patients stated that in the presence of DM, activity of alanine aminotransferase (ALT), alkaline phosphatase (AP) and gamma-glutamyl transferase (GGT) is often increased, and in some cases, prerenal azotaemia can be found as a result of dehydration.^11^ Unfortunately, the primary reference was not provided in this publication. Otherwise, information on laboratory findings in diabetic cats can only be found in veterinary textbooks.^12–14^ Here, it is described that haematological abnormalities are usually mild and include non-regenerative normochromic anaemia or stress leukogram. Common biochemical abnormalities include up to a five-fold increase in ALT, up to a three-fold increase in AP, up to a two-fold increase in bilirubin concentration (BILI) and up to a three-fold increase of concentration of cholesterol (CHOL) and triglycerides (TRI). It is also reported that creatinine and urea are expected to be normal in uncomplicated DM. Again, original references were not provided in those textbooks.^12–14^ The description of clinicopathological findings in D cats is therefore not supported by large studies.

The aims of our study therefore were three-fold: (1) to determine the prevalence of feline DM in a large laboratory convenience sample in Germany and evaluate age, sex and neuter status; (2) to describe the occurrence of changes in selected haematological and biochemical variables in diabetic (D) cats compared with non-diabetic (ND) cats; and (3) to compare selected haematological and biochemical variables between poorly-controlled diabetic (PD) and well-controlled diabetic (WD) cats.

## Materials and methods

### Laboratory data and inclusion criteria

This was a retrospective, cross-sectional study using data from laboratory submissions to a large commercial laboratory, Antech Lab Germany (formerly SYNLAB.vet), with five locations in Germany between 2015 and 2018. All submissions for routine haematology (ADVIA 2120i; Siemens Diagnostics) and serum biochemistry (AU 5800, AU 680; Beckman Coulter), including fructosamine (measured using the abovementioned biochemistry analysers) and total thyroxine (TT4) (IMMULITE 2000 XPi Immunoassay System; Siemens Medical Solutions Diagnostics) from cats within this period were included as long as the fructosamine measurement was available. The fructosamine measurement was part of the routine serum biochemistry profile and did not have to be requested separately by a submitting veterinarian, but cats with missing fructosamine as a result of insufficient sample material were excluded. Cats in which other blood parameters were occasionally missing (because of insufficient sample material) were included as long as the fructosamine measurement was performed. Where multiple blood samples from the same cat were submitted during the study, only the submission with the highest measurement of fructosamine was included. Age, sex and neuter status, as well as selected haematological and biochemical parameters (see below), were provided by the laboratory.

### Diagnosis of DM and glycaemic control

Cats were considered diabetic if they had fructosamine concentrations above the upper laboratory reference interval (RI) (⩾340 µmol/l). Increased fructosamine concentrations have been shown to be highly specific for DM diagnosis in cats.^15^ Information about clinical signs was not available and could not be used to make a diagnosis of DM. Although hyperglycaemia is a hallmark of DM, glucose concentration was not used to differentiate between D and ND cats in this study because of several limitations of glucose measurement. First, hyperglycaemia can occur in ND cats because of stress (including stress of blood sampling) and therefore is not a reliable marker of DM in cats. Second, D cats treated with insulin can be normoglycaemic; therefore, DM cannot be excluded based on normal glucose concentration. Finally, information on pre-analytical sample handling, such as time lapsed between blood sampling and centrifugation, was not available. A longer period between sampling and sample processing can lead to a decrease in blood glucose due to in vitro glycolysis.^16^

Cats with fructosamine concentrations >500 µmol/l were classified as PD cats, in agreement with previous studies.^12,17–22^ Data on clinical signs were not available and could therefore not be used for this classification. Glucose concentration was not used to assess the quality of glycaemic control because spot blood glucose measurements are not recommended to assess the quality of glycaemic control according to current DM management guidelines.^23,24^

### Comparison of selected laboratory parameters between D and ND, and PD and WD

The following laboratory parameters were compared between D and ND cats as well as PD and WD cats: red blood cell count (RBC); creatinine concentration (CREA); glucose concentration (GLU); AP; ALT; BILI; CHOL; TRI; and TT4. RBC rather than haematocrit (HCT) value or packed cell volume (PCV) was evaluated because those two latter parameters are affected by erythrocyte swelling during storage, which can lead to a significant increase in HCT/PCV within 12 h of blood collection.^25^ The proportion of cats with at least moderate anaemia (RBC ⩽4.21 ×10^12^/l), CREA >250 µmol/l (azotaemia indicating significant kidney disease likely associated with clinical signs, ie, International Renal Interest Society [IRIS] stage 3 or 4),^26^ ALT >455 U/l (5× upper RI of the laboratory), AP >315 U/l (5× upper RI of the laboratory), BILI ⩾35 µmol/l (indicating clinically detectable icterus)^27^ and increased TT4 (above laboratory RI, indicating uncontrolled hyperthyroidism) was compared between PD and WD. The cut-off of 4.21 ×10^12^/l RBCs for the diagnosis of moderate anaemia was calculated using the following equation:

$$RBC\;(\times 10^{12}/l)=\frac{HCT(\%)\;\times \;10}{MCV(fl)}$$,

where MCV is mean cell volume, and a MCV of 47.5 fl and HCT of 20%, indicating moderate anaemia, were assumed.^28^ Reticulocyte count was only available in less than 50% of the submissions; therefore, further classification of anaemia (regenerative vs non-regenerative) was not attempted.

### Statistical analysis

The statistical analysis was carried out using SPSS version 28.0 (IBM). The data were assessed for normality by visual inspection of histograms. Graphical inspection was preferred over formal statistical tests in the evaluation of normal distribution since it avoids the pitfalls of misusing the *P* values in formal tests, especially in large sample sizes.^29^ As most data were not normally distributed, the data are reported as median (interquartile range [IQR]). The prevalence of DM is reported as the proportion (in %) of diabetic cats among laboratory submissions and 95% confidence interval (CI). The Mann–Whitney U-test was applied to compare the age (D vs ND) and laboratory parameters between the different groups (D vs ND, WD vs PD). Categorical variables (sex, neuter status, proportions of cats with anaemia and elevation of selected laboratory parameters above a certain cut-off as described above) were compared using the χ^2^ test. *P* <0.05 was considered significant. A Bonferroni correction was applied to the *P* value when assessing the differences in sex and neutering status between D and ND cats because these variables are not independent from each other. To assess the effect size, r was determined following the Mann–Whitney U-tests^30^ and Cramér V following the χ^2^ test.^31^ With a large sample size, as in this study, minimal differences between study groups/populations might be significant based on *P* value, and effect sizes r or Cramér V help interpret the relevance of any significant results.^32^ According to Cohen, r or V of 0.1, 0.3 and 0.5 indicate a small, medium and large effect, respectively.^33–35^ For numerical variables, besides the *P* value and the effect size, the medians in relation to the laboratory RI were also used to interpret the results.

## Results

### Age, sex and neuter status

**Figure 1:**
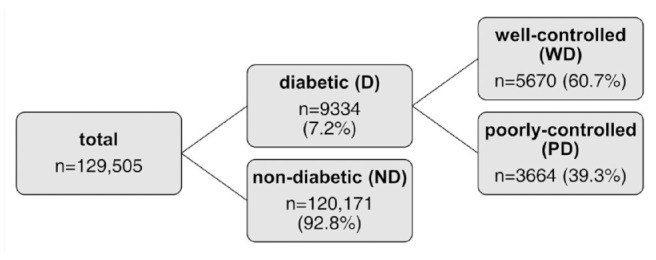
Classification of cats based on the presence or absence of diabetes (cats with fructosamine concentrations ⩾340 μmol/l were classified as diabetic [D]) and within the diabetic population into well-controlled diabetic (WD) and poorly-controlled diabetic (PD) individuals (cats with fructosamine concentrations >500 μmol/l were considered poorly controlled)

Fructosamine concentration was missing in 426 cats; therefore, 129,505 cats were included in the analysis. Of these, 9334 cats were diabetic, resulting in a DM prevalence of 7.2% (95% CI 7.0–7.4) in this population of cats based on laboratory submissions (Figure 1).

**Figure 2:**
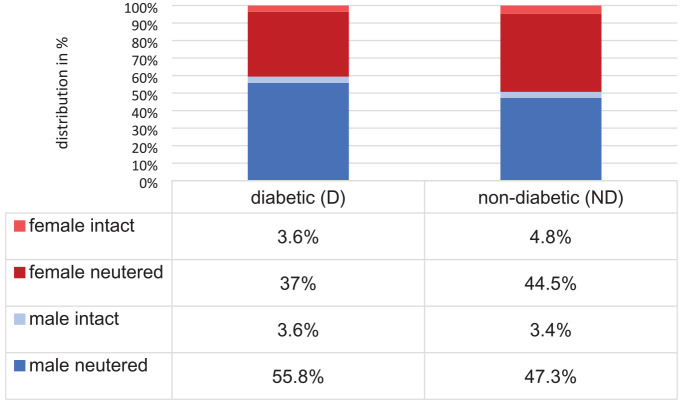
Distribution (in %) of sex and neutering status in diabetic (D) (n = 9334) and non-diabetic (ND) (n = 120,171) cats

Despite equal medians (D: 12 years, IQR 9–14; ND: 12 years, IQR 9–15), there was a significant difference in the age of D and ND cats (*P* <0.001). However, based on r = 0.02, indicating a negligible effect size, this difference was considered not relevant. Most cats in both groups were aged ⩾9 years (D: 6490 [79.8%]; ND: 83,201 [78.3%]). Information regarding sex was provided in 118,668 (91.6%) cats (60,897 male [51.3%], 57,771 female [48.7%]). At the time of blood testing, 56,834 (93.3%) male cats and 52,225 (90.4%) female cats were neutered. The distribution of sexes, including neuter status, in D and ND cats is presented in Figure 2.

The results of the statistical analysis comparing the proportions of sexes, including neuter status, between D and ND cats are shown in Table 1. Although there was a significantly higher proportion of male cats among the D cats than the ND cats, as well as a lower proportion of female intact cats and a higher proportion of male neutered cats (Figure 2, Table 1), these differences were of negligible effect sizes and therefore not considered relevant.

**Table 1:** Comparison of sex and neuter status between diabetic (D) and non-diabetic (ND) cats

Comparison between D and ND cats	Corrected *P* value according to Bonferroni	Effect size Cramér V*
1 Total male vs total female	<0.001	0.043
2 Male intact vs female intact	<0.001	0.039
3 Male neutered vs female neutered	<0.001	0.045
4 Male neutered vs male intact	0.26	0.008
5 Female neutered vs female intact	0.77	0.006

*P* <0.05 was considered statistically significant

* Cramér V: 0.1 = small effect; 0.3 = medium effect; 0.5 = large effect

### Comparison of selected laboratory parameters between D and ND cats

Significant differences between D and ND cats were detected for all selected haematological and biochemical parameters based on a *P* value <0.05 (Table 2).

**Table 2:** Comparison of selected laboratory parameters between diabetic (D) and non-diabetic (ND) cats

Parameter	Number (n)	Laboratory RI	D cats	ND cats	*P* value	Effect size r*
RBC (T/l)	Total: 127,575 D: 9127 ND: 118,448	5.0–10.0	8.86 (7.75– 9.89)	8.62 (7.56– 9.57)	<0.001	0.043
CREA (µmol/l)	Total: 129,452 D: 9312 ND: 120,140	0–168	123.8 (99– 159)	130 (105.2–162.7)	<0.001	0.029
AP (U/l)	Total: 128,761 D: 9201 ND: 119,560	<63	47 (28– 77)	33 (23–49)	<0.001	0.11
ALT (U/l)	Total: 129,314 D: 9283 ND: 120,031	<91	79 (55–133)	66 (49–100)	<0.001	0.075
TT4 (nmol/l)	Total:120,430 D:7894 ND:112,536	12.9–51.5	18.66 (11.7–27.28)	22.52 (15.19–32.18)	<0.001	0.080
BILI (µmol/l)	Total: 123,789 D: 8488 ND: 115,301	<8.55	3.42 (3.3–5.13)	3.42 (2.91– 3.42)	<0.001	0.094
CHOL (mmol/l)	Total: 129,468 D: 9325 ND: 120,143	1.81–3.88	6.12 (4.77–8.02)	4.69 (3.76– 5.85)	<0.001	0.18
TRI (mmol/l)	Total: 129,414 D: 9305 ND: 120,109	0.23–1.23	0.96 (0.59–2.02)	0.59 (0.43– 0.87)	<0.001	0.18
GLU (mmol/l)	Total: 106,014 D: 7098 ND: 98,916	3.9–8.3	12.93 (5.94–23.2)	5.67 (4.77– 7.09)	<0.001	0.23

Data are median (IQR) unless otherwise indicated. *P* <0.05 was considered statistically significant

* r = 0.1 small effect, r = 0.3 medium effect, r = 0.5 large effect.

ALT = alanine aminotransferase; AP = alkaline phosphatase; BILI = bilirubin; CHOL = cholesterol; CREA = creatinine; D = diabetic, GLU = glucose; IQR = interquartile range; ND = non-diabetic; RBC = red blood cell count; RI = reference interval; TT4 = total thyroxine; TRI = triglycerides

RBC, AP, ALT, CHOL, TRI and GLU were higher in the D cats, while CREA and TT4 were lower. However, for all tested variables apart from CHOL and GLU, the medians lay within the RI in both groups. The medians of CHOL were outside (above) the laboratory RI in both groups and the median of GLU was above the laboratory RI in the D. Furthermore, the effect sizes were negligible (r <0.1) for all parameters but AP, CHOL, TRI and GLU (Table 2). In these four, r in the range of 0.1–0.3 indicated a meaningful but small relevance. Taken together, the most significant and relevant finding was the higher CHOL in D cats when compared with ND cats. As expected, GLU was higher in D cats when compared with ND cats.

### Comparison of selected laboratory parameters between WD and PD

Of the diabetic patients, 60.7% (n = 5670, 95% CI 59.8–61.7) were well controlled and 39.3% (n = 3664, 95% CI 38.3–40.3) were poorly controlled based on a fructosamine concentration ⩽500 μmol/ or >500 μmol/l, respectively. Similar to the comparison between D and ND cats, significant differences between WD and PD were detected for all selected laboratory parameters based on *P* <0.05 (Table 3).

**Table 3:** Comparison of selected laboratory parameters between well-controlled diabetic (WD) and poorly-controlled diabetic (PD) cats with effect size r

Parameter	Number (n)	Laboratory RI	WD	PD	*P* value	Effect size r*
RBC (T/l)	Total: 9127 WD: 5549 PD: 3578	5.0–10.0	8.76 (7.61– 9.81)	9.01 (8–9.98)	<0.001	0.087
CREA (µmol/l)	Total: 9312 WD: 5680 PD: 3632	0–168	132.6 (105.2–173.9)	112.3 (92.8–137.9)	<0.001	0.23
AP (U/l)	Total: 9201 WD: 5597 PD: 3604	<63	35 (23–59)	66 (47–92)	<0.001	0.39
ALT (U/l)	Total: 9283 WD: 5676 PD: 3607	<91	73 (53–119)	90 (61–156)	<0.001	0.14
TT4 (nmol/l)	Total: 7894 WD: 4962 PD: 2932	12.9–51.5	21.49 (13.51– 30.5)	14.93 (10.23–21.2)	<0.001	0.26
BILI (µmol/l)	Total: 8488 WD: 5046 PD: 3442	<8.55	3.42 (3–4.96)	3.5 (3.42–5.13)	<0.001	0.11
CHOL (mmol/l)	Total: 9325 WD: 5688 PD: 3637	1.81–3.88	5.44 (4.32– 6.97)	7.46 (5.85– 9.32)	<0.001	0.39
TRI (mmol/l)	Total: 9305 WD: 5680 PD: 3625	0.23–1.23	0.775 (0.5– 1.35)	1.44 (0.84– 3.66)	<0.001	0.35
GLU (mmol/l)	Total: 7098 WD: 4286 PD: 2812	3.9–8.3	6.89 (5–11.31)	23.7 (20.15–27.3)	<0.001	0.69

Data are median (IQR) unless otherwise indicated; *P* <0.05 was considered statistically significant

* r = 0.1 small effect, r = 0.3 medium effect, r = 0.5 large effect

ALT = alanine aminotransferase; AP = alkaline phosphatase; BILI = bilirubin; CHOL = cholesterol; CREA = creatinine; GLU = glucose; IQR = interquartile range; RBC = red blood cell count; RI = reference interval; TT4 = total thyroxine; TRI = triglycerides

RBC, AP, ALT, BILI, CHOL, TRI and GLU were higher and CREA and TT4 were lower in PD compared with WD. Again, most medians lay within the RI, but CHOL in both groups and AP, TRI and GLU in PD were increased (ie, above the upper limit of the RI). Based on effect sizes, the difference in CREA, ALT, TT4 and BILI had a small effect, while the difference in AP, CHOL and TRI had a medium effect and the difference in GLU had a large effect. Based on the effect sizes and medians outside of the RI, the higher AP, CHOL, TRI and GLU in PD when compared with WD were considered the most relevant findings.

### Frequencies of selected laboratory abnormalities between WD and PD

Frequencies of at least moderate anaemia, azotaemia indicating significant kidney disease (IRIS stage 3 or 4), significant increase in ALT and AP activities (at least five-fold), hyperbilirubinaemia associated with clinically detectable icterus and increased TT4 indicating uncontrolled hyperthyroidism in WD and PD are summarised in Table 4.

**Table 4:** Frequencies of at least moderate anaemia, azotaemia, increase in liver enzyme activities, hyperbilirubinaemia, indicating clinically detectable icterus, and increased total thyroxine, indicating uncontrolled hyperthyroidism in well-controlled diabetic (WD) and poorly-controlled diabetic (PD) cats with effect size Cramér V

Parameter	Laboratory RI	WD	PD	*P* value	Cramér V*
RBC ⩽4.21 (T/l) = moderate anaemia	5.0–10.0	5523 (1.5)	3604 (0.4)	<0.001	0.052
CREA >250 (µmol/l) = IRIS 3+4	0–168	5654 (13.4)	3658 (4.7)	<0.001	0.14
AP >315 (U/l) = five-fold increase	<63	5571 (3.1)	3630 (1.1)	<0.001	0.065
ALT >455 (U/l) = five-fold increase	<91	5650 (6)	3633 (4.7)	0.008	0.027
BILI >35 (µmol/l) = clinical icterus	<8.55	5022 (7.3)	3466 (3.0)	<0.001	0.092
TT4 >51.5 (nmol/l)	12.9–51.5	5379 (5.6)	3593 (1.2)	<0.001	0.109

Data are n (%) unless otherwise indicated; *P* <0.05 was considered statistically significant

* Cramér V: 0.1 small effect, 0.3 medium effect, 0.5 large effect

ALT = alanine aminotransferase; AP = alkaline phosphatase; BILI = bilirubin; CREA = creatinine; IRIS = International Renal Interest Society; RBC = red blood cell count; RI = reference interval; TT4 = total thyroxine

All abnormalities were more frequently present in WD; however, although significant differences were detected between WD and PD based on *P*<0.05, Cramér V indicated negligible relevance for all parameters but azotaemia and proportion of cats with increased TT4 (both small effect).

## Discussion

In this large laboratory convenience sample in Germany, 7.2% of cats were diabetic. As this is the first large-scale study evaluating laboratory abnormalities in diabetic cats, this finding cannot be directly compared with other studies, interrogating different populations. Other investigators examined insurance records^1^ and data from primary care practices,^2^ large feline-only clinics^4^ or veterinary teaching hospitals,^3,5^ where 0.08–1.24% of cats were diabetic. None of the studies perfectly represent the population of diabetic cats in a particular geographic region or country. However, nationwide registers of diabetic animals do not exist and therefore information is currently only available for those abovementioned subpopulations.

The median age of both diabetic and non-diabetic cats was 12 years, indicating that the results reflect a prevalence of DM within the older population rather than the general cat population in Germany. As age is a well-known risk factor for DM,^2,4–8^ a higher prevalence is expected in older cats. Although some blood samples might have been submitted for health checks, a recent study revealed that blood tests are not routinely performed as a part of preventive healthcare in German veterinary practices.^36^ It is therefore likely that the majority of laboratory submissions are from sick animals. Older cats are more likely to suffer from diseases, explaining the higher age of population examined in the present study. The prevalence of DM might have been lower if younger cats were included; however, DM is a multifactorial disease potentially influenced by the presence of comorbidities^37^ and can therefore occur at any age.

The median age of diabetic cats in this study population, 12 years, is comparable to previous reports and further supports increasing age as a risk factor.^2,4–8^ There were more males (5071, 59.4%) than females (3470, 40.6%) within the diabetic study population; however, based on Cramér V, this difference was of negligible effect size and therefore not relevant. This is in contrast to various previous reports, where male sex was identified as a risk factor for DM in cats.^1,3,5,6,8–10^ To the authors’ knowledge, Cramér V was not determined previously; however, in most reports, the percentages of male diabetic cats were in the range of 62.6–85%, considerably higher than in our study.^1,3,5,10^

Regarding the effect of neuter status on diabetes risk, contradictory information exists. While some studies found an increased risk of DM in neutered cats regardless of sex,^1^ others did not.^5^ In the present study, neutered cats were not significantly overrepresented among diabetics. Weight gain after neutering^38–40^ has been discussed as the reason for an increased risk of DM in neutered animals, with obesity being a well-known risk factor for DM.^1–3,5,10^ In a small experimental study, however, although neutered animals gained weight, this had minimal effects on the indices of glucose tolerance,^41^ indicating that neutering alone might not substantially impact the risk of DM development. Indeed, multiple risk factors for DM in cats exist, including obesity^2,5,6,10^ and older age,^5,6^ as discussed above, as well as reduced physical activity/indoor confinement^1,10^ and in some studies also feeding dry food.^10^ These factors likely interact; therefore, it is difficult to estimate the impact of a single variable such as sex on DM risk in an individual cat.

Another objective of this study was to describe clinicopathological abnormalities in diabetic cats and their association with poorly controlled DM. As expected, glucose concentration was higher in D cats when compared with ND cats, and in PD when compared with WD. Although significant differences between D and ND as well as between WD and PD were also detected for the remaining tested variables, few differences were considered relevant based on effect sizes and medians outside (above) the RI. PD cats had increased CHOL, TRI and AP, with medium effects sizes, indicating a relevant difference between PD and WD. Although the median for CHOL lay above RI in ND cats too, TRI and AP were not increased in this group. The reason for increased CHOL in ND cats cannot be established from a retrospective analysis of laboratory data, but the combination of increased CHOL, TRI and AP in PD cats could suggest the presence of hepatic lipidosis,^12^ which has been described to occur in DM cats.^42^ This combination of laboratory findings has been described in the literature to commonly occur in diabetic cats,^9,11–14^ but their association with poor diabetic control has not been reported; however, to the authors’ knowledge, this has not yet been specifically examined. On the other hand, no clinically relevant increase in bilirubin was detected in this study. Although hyperbilirubinaemia in addition to increased AP and/or ALT is a common finding in hepatic lipidosis, its absence does not exclude the disease.^42^ Interestingly, in one study, diabetic cats with higher cholesterol were also less likely to develop diabetic remission, which fits with our findings of higher cholesterol in PD cats.^43^

An interesting finding in this study was the increased frequency of azotaemia (creatinine >250 µmol/l, indicating IRIS stage 3 or higher)^26^ in WD compared with PD cats. In humans, so-called diabetic nephropathy with proteinuria is one of the feared complications of DM.^44,45^ Currently, there is no evidence for diabetic nephropathy in cats,^46^ and a clear link between DM and chronic kidney disease (CKD) could not be established in previous investigations.^46,47^ One study identified shorter survival in diabetic cats with higher creatinine concentrations at DM diagnosis.^48^ In the present study using laboratory submissions, the time of DM diagnosis and survival was unknown and the reason for the higher proportion of cats with azotaemia in WD in comparison with PD could not be determined. Future studies are needed to assess this finding, ideally in a prospective manner.

Medians of TT4 were within the RI in both PD and WD cats, but 5.6% and 1.2% of cats with WD and PD, respectively, had TT4 above the RI, indicating uncontrolled hyperthyroidism. Other studies reported that approximately 4.5% of diabetic cats suffer from concurrent hyperthyroidism,^49^ which is similar to this investigation. However, because of the study design (lack of clinical information), we were only able to detect cats with uncontrolled hyperthyroidism. Cats with hyperthyroidism controlled by antithyroid drugs or other treatment options could not be identified, likely underestimating the true prevalence of hyperthyroidism in this population of D cats based on laboratory submissions. The lower proportion of cats with uncontrolled hyperthyroidism among the PD cats might also be the result of the effect of non-thyroidal illness (ie, DM in this study).^50^

The main limitation of this study is its retrospective character and the use of laboratory data with no clinical information available. The diagnosis of DM therefore relied on serum fructosamine alone, and this parameter can be falsely decreased in cats with hypoproteinaemia^51^ or hyperthyroidism,^52^ and can also be within RI in diabetic cats with tight glycaemic control.^37^ In dogs, fructosamine concentration can be falsely increased in hypothyroidism,^53^ but this condition is extremely rare in cats^54^ and it is unlikely that it has had any meaningful impact on the results of the present study. Other conditions leading to falsely increased fructosamine concentrations have not been identified by a search of the literature. Given the large sample size in our study, the number of falsely classified cats is likely negligible. Unfortunately, as a result of the effect of stress, hyperglycaemia is not a reliable indicator of DM in cats,^55^ and could not be used to identify diabetic cats misclassified by fructosamine.

A further limitation is that glycaemic control could only be assessed on the basis of fructosamine concentration. This would ideally be judged based on assessment of clinical signs;^23,24^ however this information was not available because of the study design. A single study using laboratory submissions to assess feline pancreatic lipase (fPLI) in diabetic cats also made use of fructosamine to assess the quality of glycaemic control.^56^ Although that study attempted to obtain clinical information using questionnaires, such information could only be obtained in a proportion of cats. In the present study, information on clinical signs could not be acquired as anonymised data was provided by Antech Lab Germany, in alignment with data protection, therefore submitting veterinarian or cat owners could not be contacted. In addition, contacting over 100,000 veterinarians or owners would not have been possible and information collected retrospectively might not have been recalled correctly.

Another limitation is that it is not known whether cats were fasted before blood sampling and postprandial hyperlipidaemia might have affected the results. This might have contributed to hypercholesterolaemia detected in both D and ND cats; however, postprandial hypertriglyceridaemia would also be expected,^57^ but was not present in this study, making the effect of feeding less likely. Furthermore, diseases other than DM might have impacted the observed laboratory changes (CHOL, TRI and AP). However, the diseases that might impact those parameters in cats are either rare (hypothyroidism, Cushing’s disease)^57,58^ or uncommon (cholangiohepatitis)^59^ and therefore unlikely to have occurred at any higher frequency in the present study. The effect of hepatic lipidosis secondary to disease other than DM cannot be excluded.

In addition, given the cross-sectional study design and lack of clinical data, the effect of prerenal causes (ie, dehydration) on creatinine concentration cannot be ruled out, and the cut-offs were purely chosen based on their clinical utility and not to claim that the cats truly had IRIS 3 or 4 CKD. For staging, at least two creatinine values in a stable, well-hydrated patient would have been needed.^26^

## Conclusions

To the authors’ knowledge, the present study is the first large investigation of DM-associated laboratory changes in a large laboratory convenience sample. The age of German diabetic cats (median 12 years) in this study using laboratory submissions was similar to other previously reported populations, but male and neutered cats were not overrepresented. Similar to information provided in book chapters based mainly on experts’ opinions, clinicopathological abnormalities in diabetic cats identified in the present large-scale study were mild. The most relevant findings were hypercholesterolaemia, hypertriglyceridaemia and increased AP in PD cats, which could suggest the presence of hepatic lipidosis in this group. Laboratory reassessment might be indicated after the stabilisation of DM, because any clinicopathological changes were more common in poorly-controlled diabetic cats.

## References

[1] McCann TM Simpson KE Shaw DJ , et al. Feline diabetes mellitus in the UK: the prevalence within an insured cat population and a questionnaire-based putative risk factor analysis. J Feline Med Surg 2007; 9: 289–299. 17392005 10.1016/j.jfms.2007.02.001 PMC10822632

[2] O’Neill DG Gostelow R Orme C , et al. Epidemiology of diabetes mellitus among 193,435 cats attending primary-care veterinary practices in England. J Vet Intern Med 2016; 30: 964–972. 27353396 10.1111/jvim.14365 PMC5094533

[3] Sallander M Eliasson J Hedhammar A. Prevalence and risk factors for the development of diabetes mellitus in Swedish cats. Acta Vet Scand 2012; 54. DOI: 10.1186/1751-0147-54-61. 10.1186/1751-0147-54-61 PMC3537597 23114390

[4] Lederer R Rand JS Jonsson NN , et al. Frequency of feline diabetes mellitus and breed predisposition in domestic cats in Australia. Vet J 2009; 179: 254–258. 18155627 10.1016/j.tvjl.2007.09.019

[5] Prahl A Guptill L Glickman NW , et al. Time trends and risk factors for diabetes mellitus in cats presented to veterinary teaching hospitals. J Feline Med Surg 2007; 9: 351–358. 17449313 10.1016/j.jfms.2007.02.004 PMC10832956

[6] Panciera DL Thomas CB Eicker SW , et al. Epizootiologic patterns of diabetes mellitus in cats: 333 cases (1980–1986). J Am Vet Med Assoc 1990; 197: 1504–1508. 2272886

[7] Rand JS Bobbermien LM Hendrikz JK , et al. Over representation of Burmese cats with diabetes mellitus. Aust Vet J 1997; 75: 402–405. 9247686 10.1111/j.1751-0813.1997.tb14340.x

[8] Öhlund M Fall T Ström Holst B , et al. Incidence of diabetes mellitus in insured Swedish cats in relation to age, breed and sex. J Vet Intern Med 2015; 29: 1342–1347. 26179258 10.1111/jvim.13584 PMC4858030

[9] Crenshaw KL Peterson ME. Pretreatment clinical and laboratory evaluation of cats with diabetes mellitus: 104 cases (1992–1994). J Am Vet Med Assoc 1996; 209: 943–949. 8790546

[10] Öhlund M Egenvall A Fall T , et al. Environmental risk factors for diabetes mellitus in cats. J Vet Intern Med 2017; 31: 29–35. 27906456 10.1111/jvim.14618 PMC5259626

[11] Metzger FL Rebar AH. Clinical pathology interpretation in geriatric veterinary patients. Vet Clin North Am Small Anim Pract 2012; 42: 615–629. 10.1016/j.cvsm.2012.04.004 22720804

[12] Feldman EC Nelson RW Reusch C , et al (eds). Canine and feline endocrinology. 4th ed. London: Elsevier Health Sciences, 2014.

[13] Ettinger SJ . Textbook of veterinary internal medicine diseases of the dog and the cat. 8th ed. St Louis, MO: Elsevier, 2017.

[14] Feldman EC Fracassi F Peterson ME (eds). Feline endocrinology. Milano: Edra, 2019.

[15] Plier ML Grindem CB MacWilliams PS , et al. Serum fructosamine concentration in nondiabetic and diabetic cats. Vet Clin Pathol 1998; 27: 34–39. 12075546 10.1111/j.1939-165x.1998.tb01013.x

[16] Sacks DB Bruns DE Goldstein DE , et al. Guidelines and recommendations for laboratory analysis in the diagnosis and management of diabetes mellitus. Clinical Chemistry 2002; 48: 436–472. 11861436

[17] Thoresen SI Bredal WP. Clinical usefulness of fructosamine measurements in diagnosing and monitoring feline diabetes mellitus. J Small Anim Pract 1996; 37: 64–68. 8656595 10.1111/j.1748-5827.1996.tb01940.x

[18] Weaver KE Rozanski EA Mahony OM , et al. Use of glargine and lente insulins in cats with diabetes mellitus. J Vet Intern Med 2006; 20: 234. 16594577 10.1892/0891-6640(2006)20[234:uogali]2.0.co;2

[19] Boari A Aste G Rocconi F , et al. Glargine insulin and high-protein-low-carbohydrate diet in cats with diabetes mellitus. Vet Res Commun 2008; 32 Suppl 1: S243–S245. 10.1007/s11259-008-9119-x 18685984

[20] Nelson RW Henley K Cole C. Field safety and efficacy of protamine zinc recombinant human insulin for treatment of diabetes mellitus in cats. J Vet Intern Med 2009; 23: 787–793. 19566845 10.1111/j.1939-1676.2009.0342.x

[21] Linari G Fleeman L Gilor C , et al. Insulin glargine 300 U/ml for the treatment of feline diabetes mellitus. J Feline Med Surg 2022; 24: 168–176. 34009061 10.1177/1098612X211013018 PMC10812176

[22] Benedict SL Mahony OM McKee TS , et al. Evaluation of bexagliflozin in cats with poorly regulated diabetes mellitus. Can J Vet Res 2022; 86: 52–58. 34975223 PMC8697324

[23] Sparkes AH Cannon M Church D , et al. ISFM consensus guidelines on the practical management of diabetes mellitus in cats. J Feline Med Surg 2015; 17: 235–250. 25701862 10.1177/1098612X15571880 PMC11148891

[24] Behrend E Holford A Lathan P , et al. 2018 AAHA diabetes management guidelines for dogs and cats. J Am Anim Hosp Assoc 2018; 54: 1–21. 29314873 10.5326/JAAHA-MS-6822

[25] Bauer N Nakagawa J Dunker C , et al. Evaluation of the automated hematology analyzer Sysmex XT-2000iV™ compared to the ADVIA ® 2120 for its use in dogs, cats, and horses. Part II: accuracy of leukocyte differential and reticulocyte count, impact of anticoagulant and sample aging. J Vet Diagn Invest 2012; 24: 74–89. 22362937 10.1177/1040638711436243

[26] International Renal Interest Society. IRIS staging of CKD. http://www.iris-kidney.com/pdf/IRIS_Staging_of_CKD_modified_2019.pdf (modified 2019, accessed 8 December 2022).

[27] Sherding RG. Feline jaundice. J Feline Med Surg 2000; 2: 165–169. 11716613 10.1053/jfms.2000.0088 PMC10829111

[28] Stockham SL Scott MA . Chapter 3: Erythrocytes. In: Fundamentals of veterinary clinical pathology. 2nd ed. Ames, IA: Blackwell Publishing, 2008; pp 107–223.

[29] Cohen J Cohen P . Applied multiple regression analysis for the behavioral sciences. 2nd ed. Hillsdale, NJ: Lawrence Erlbaum Associates, 1975.

[30] Rosenthal R. Meta-analytic procedures for social research. Newbury Park, CA: Sage Publications, 1991.

[31] Tomczak M Tomczak E. The need to report effect size estimates revisited. An overview of some recommended measures of effect size. Trends Sport Sci 2014; 1: 19–25.

[32] Lin M Lucas HC Shmueli G. Research commentary – too big to fail: large samples and the p-value problem. Info Sys Res 2013; 24: 906–917.

[33] Cohen J. Statistical power analysis for the behavioral sciences. 2nd ed. Hillsdale, NJ: Erlbaum, 1988.

[34] Cohen J. A power primer. Psychol Bull 1992; 112: 155–159. 19565683 10.1037//0033-2909.112.1.155

[35] Ellis PD. The essential guide to effect sizes. Cambridge: Cambridge University Press, 2012.

[36] Klinger C , et al. Kleintiermedizin in Deutschland – Analyse des Fallaufkommens. Der Praktische Tierarzt 2016: 774–787.

[37] Niessen SJM Bjornvad C Church DB , et al. Agreeing Language in Veterinary Endocrinology (ALIVE): diabetes mellitus – a modified Delphi-method-based system to create consensus disease definitions. Vet J 2022; 289. DOI: 10.1016/j.tvjl.2022.105910. 10.1016/j.tvjl.2022.105910 36182064

[38] Courcier EA O’Higgins R Mellor DJ , et al. Prevalence and risk factors for feline obesity in a first opinion practice in Glasgow, Scotland. J Feline Med Surg 2010; 12: 746–753. 20685143 10.1016/j.jfms.2010.05.011 PMC11135528

[39] Nguyen PG Dumon HJ Siliart BS , et al. Effects of dietary fat and energy on body weight and composition after gonadectomy in cats. Am J Vet Res 2004; 65: 1708–1713. 15631038 10.2460/ajvr.2004.65.1708

[40] Martin L Siliart B Dumon H , et al. Leptin, body fat content and energy expenditure in intact and gonadectomized adult cats: a preliminary study. J Anim Physiol Anim Nutr (Berl) 2001; 85: 195–199. 11686788 10.1046/j.1439-0396.2001.00322.x

[41] Fettman MJ Stanton CA Banks LL , et al. Effects of neutering on bodyweight, metabolic rate and glucose tolerance of domestic cats. Res Vet Sci 1997; 62: 131–136. 9243711 10.1016/s0034-5288(97)90134-x

[42] Center SA Crawford MA Guida L , et al. A retrospective study of 77 cats with severe hepatic lipidosis: 1975–1990. J Vet Intern Med 1993; 7: 349–359. 8114031 10.1111/j.1939-1676.1993.tb01030.x

[43] Zini E Hafner M Osto M , et al. Predictors of clinical remission in cats with diabetes mellitus. J Vet Intern Med 2010; 24: 1314–1321. 20840299 10.1111/j.1939-1676.2010.0598.x

[44] Zhang X-X Kong J Yun K. Prevalence of diabetic nephropathy among patients with type 2 diabetes mellitus in China: a meta-analysis of observational studies. J Diabetes Res 2020; 2020. DOI: 10.1155/2020/2315607. 10.1155/2020/2315607 PMC7023800 32090116

[45] Ritz E Rychlík I Locatelli F , et al. End-stage renal failure in type 2 diabetes: a medical catastrophe of worldwide dimensions. Am J Kidney Dis 1999; 34: 795–808. 10561134 10.1016/S0272-6386(99)70035-1

[46] Zini E Benali S Coppola L , et al. Renal morphology in cats with diabetes mellitus. Vet Pathol 2014; 51: 1143–1150. 24565829 10.1177/0300985813516645

[47] Greene JP Lefebvre SL Wang M , et al. Risk factors associated with the development of chronic kidney disease in cats evaluated at primary care veterinary hospitals. J Am Vet Med Assoc 2014; 244: 320–327. 24432964 10.2460/javma.244.3.320

[48] Callegari C Mercuriali E Hafner M , et al. Survival time and prognostic factors in cats with newly diagnosed diabetes mellitus: 114 cases (2000–2009). J Am Vet Med Assoc 2013; 243: 91–95. 23786195 10.2460/javma.243.1.91

[49] Schaefer S Kooistra HS Riond B , et al. Evaluation of insulin-like growth factor-1, total thyroxine, feline pancreas-specific lipase and urinary corticoid-to-creatinine ratio in cats with diabetes mellitus in Switzerland and the Netherlands. J Feline Med Surg 2017; 19: 888–896. 27578200 10.1177/1098612X16664390 PMC11104121

[50] Carney HC Ward CR Bailey SJ , et al. 2016 AAFP guidelines for the management of feline hyperthyroidism. J Feline Med Surg 2016; 18: 400–416. 27143042 10.1177/1098612X16643252 PMC11132203

[51] Reusch CE Haberer B. Evaluation of fructosamine in dogs and cats with hypo- or hyperproteinaemia, azotaemia, hyperlipidaemia and hyperbilirubinaemia. Vet Rec 2001; 148: 370–376. 11321552 10.1136/vr.148.12.370

[52] Reusch CE Tomsa K. Serum fructosamine concentration in cats with overt hyperthyroidism. J Am Vet Med Assoc 1999; 215: 1297–1300. 10553441

[53] Reusch CE Gerber B Boretti FS. Serum fructosamine concentrations in dogs with hypothyroidism. Vet Res Commun 2002; 26: 531–536. 12416867 10.1023/a:1020287430949

[54] Peterson ME Carothers MA Gamble DA , et al. Spontaneous primary hypothyroidism in 7 adult cats. J Vet Intern Med 2018; 32: 1864–1873. 30294940 10.1111/jvim.15239 PMC6271337

[55] Laluha P Gerber B Laluhová D , et al. Stresshyperglykämie bei kranken Katzen: Eine retrospektive Studie über 4 Jahre. Schweiz Arch Tierheilkd 2004; 146: 375–383. 15379170 10.1024/0036-7281.146.8.375

[56] Forcada Y German AJ Noble PJM , et al. Determination of serum fPLI concentrations in cats with diabetes mellitus. J Feline Med Surg 2008; 10: 480–487. 18639478 10.1016/j.jfms.2007.04.007 PMC11271242

[57] Jordan E Kley S Le N-A , et al. Dyslipidemia in obese cats. Domest Anim Endocrinol 2008; 35: 290–299. 18692343 10.1016/j.domaniend.2008.05.008

[58] Blois SL Dickie EL Kruth SA , et al. Multiple endocrine diseases in cats: 15 cases (1997–2008). J Feline Med Surg 2010; 12: 637–642. 20580584 10.1016/j.jfms.2010.03.017 PMC10911494

[59] Callahan Clark JE Haddad JL Brown DC , et al. Feline cholangitis: a necropsy study of 44 cats (1986–2008). J Feline Med Surg 2011; 13: 570–576. 21719332 10.1016/j.jfms.2011.05.002 PMC10822413

